# Antifungal Use and Resistance in a Lower–Middle-Income Country: The Case of Lebanon

**DOI:** 10.3390/antibiotics12091413

**Published:** 2023-09-06

**Authors:** Nesrine Hassoun, Issmat I. Kassem, Monzer Hamze, Jad El Tom, Nicolas Papon, Marwan Osman

**Affiliations:** 1 Faculty of Public Health, Lebanese University, Tripoli 1300, Lebanon; nesrine.hassoun@st.ul.edu.lb; 2 Center for Food Safety, Department of Food Science and Technology, University of Georgia, 1109 Experiment Street, Griffin, GA 30223, USA; issmat.kassem@uga.edu; 3 Laboratoire Microbiologie Santé et Environnement (LMSE), Doctoral School of Sciences and Technology, Faculty of Public Health, Lebanese University, Tripoli 1300, Lebanon; mhamze@monzerhamze.com; 4 School of Pharmacy, Lebanese American University, Byblos 1401, Lebanon; jad.eltom@lau.edu; 5 University of Angers, University of Brest, IRF, SFR ICAT, F-49000 Angers, France; nicolas.papon@univ-angers.fr; 6 Cornell Atkinson Center for Sustainability, Cornell University, Ithaca, NY 14853, USA; 7 Department of Public and Ecosystem Health, College of Veterinary Medicine, Cornell University, Ithaca, NY 14853, USA

**Keywords:** antifungal resistance, surveillance, One Health, epidemiology, *Candida*, *Aspergillus*, Lebanon

## Abstract

Antimicrobial resistance is a serious threat, particularly in low- and middle-income countries (LMICs). Antifungal resistance is often underestimated in both healthcare and non-clinical settings. In LMICs, it is believed that the inappropriate use of antifungals, limited surveillance systems, and low diagnostic capacities are significant drivers of resistance. Like other LMICs, Lebanon lacks antifungal use and resistance surveillance programs, and the impact of antifungal resistance in the country remains unclear, especially during the unfolding economic crisis that has severely affected medical care and access to safe food and water. Interestingly, the widespread use of antifungals in medicine and agriculture has raised concerns about the development of antifungal resistance in Lebanon. In this light, we aimed to survey available antifungal drugs in the country and evaluate susceptibility patterns of prevalent fungal species to guide empiric treatments and develop antifungal stewardship programs in Lebanon. We noted that the economic crisis resulted in significant increases in antifungal drug prices. Additionally, a comprehensive literature search across PubMed, ScienceDirect, and Google Scholar databases identified 15 studies on fungal infections and antifungal resistance conducted from 1998 to 2023 in Lebanon. While data on antifungal resistance are limited, 87% of available studies in Lebanon focused on candidiasis, while the remaining 13% were on aspergillosis. Overall, we observed a marked antimicrobial resistance among *Candida* and *Aspergillus* species. Additionally, incidences of *Candida auris* infections have increased in Lebanese hospitals during the COVID-19 pandemic, with a uniform resistance to fluconazole and amphotericin-B. Taken together, a One Health approach, reliable diagnostics, and prudent antifungal use are required to control the spread of resistant fungal pathogens in healthcare and agricultural settings.

## 1. Introduction

Antimicrobial resistance has become a significant global public health threat, especially in low- and middle-income countries (LMICs) that are challenged with different medical, environmental, and socioeconomic problems [1,2,3]. Although bacterial resistance to antibiotics has received great attention in recent years, antifungal resistance is relatively underappreciated, and its real burden is still underestimated in both healthcare and non-clinical settings.

Fungal diseases represent a growing concern in human and veterinary medicine, with a significant impact on morbidity and mortality, particularly among predisposed patients, and substantial healthcare costs [4,5,6,7]. As observed in bacteria, fungi display primary (intrinsic) and/or secondary (acquired) drug resistance [8,9]. Concerning intrinsic resistance, a series of prominent pathogenic fungal species are well known for being less susceptible to one or several antifungal compounds, such as the yeast *Pichia kudriavzevii* (formerly known as *Candida krusei*) towards fluconazole [10] and the filamentous fungi *Aspergillus terreus* towards amphotericin-B [11]. In addition, the capacity of fungi to acquire antifungal resistance mainly relies on their genome plasticity, which allows these microorganisms to evolve and adapt dynamically to environmental cues. Upon antifungal exposure, some mutations, underlying changes in gene expression, and/ or modification of protein properties can indeed drive the emergence of secondary resistance [12]. For example, resistance to azoles is associated with three major mechanisms: (i) increased expression of the azole target gene, (ii) changes in the amino-acid sequence of the azole target at the level of the azole binding site, and (iii) increased production of multidrug efflux transporters [13,14].

It is known that the inappropriate use of antifungals poses a selective pressure that is a key driver for the emergence and dissemination of antifungal resistance in the human–animal–environment continuum [6,15,16,17,18]. This is not surprising, because antifungals are used in clinical settings, as well as in agriculture for controlling animal and plant diseases and increasing crop yields [19,20]. Notably, approximately half of veterinary and agriculture antifungal drugs are also approved for use in human medicine [21]. For example, triazole, a common class of antifungal agents commonly used to treat invasive fungal infections in humans, is also among the most used in agriculture [20]. Consequently, the efficacy of antifungal drugs to treat human infections is being affected by the emergence of antifungal-resistant determinants in agriculture, including pathogens/microorganisms associated with many animals and plants and their environment [22]. Hence, it is critical to tackle antifungal resistance using a One Health approach, which acknowledges the interconnectedness of the health of humans, animals, plants, and environment

In 2017, Lebanon joined the World Health Organization’s (WHO) Global Antimicrobial Resistance Surveillance System (GLASS) [23,24]. However, the scope of this collaborative platform does not currently encompass antifungal resistance. Additionally, there is currently no nationwide system in place that implements a comprehensive antifungal use and resistance surveillance program in the country. Altogether, the full extent of the impact of antifungal use and resistance remains largely unknown. This is highly relevant, because Lebanon has been encountering an array of serious challenges that are posing significant threats to its social and economic stability. These include the massive influx of refugees (~1.5 million) since 2011, as well as an unprecedented economic collapse since October 2019, which has resulted in hyperinflation, high unemployment rates, major strikes in the public sector, and a steep decline in the value of the Lebanese currency. These conditions resulted, in turn, in a severe shortage in importation (Lebanon relies heavily on imports to meet its nutritional and medical needs), reducing the availability and increasing the prices of essential items that impact public health and agriculture. Moreover, the COVID-19 pandemic has magnified these issues, increasing the surge in poverty rates and overwhelming the country’s healthcare system further. Taken together, these conditions are very suitable for the emergence and re-emergence of disease, especially in the most vulnerable populations. Despite these challenges and the increase in antimicrobial drug prices, anecdotal evidence suggests that the inappropriate use of antimicrobials, including antifungals, is still notable in human medicine and the agriculture sector in Lebanon. The relatively low cost of antimicrobial drugs compared to healthcare or agriculture services and other treatment modalities associated with poor knowledge often prompts individuals to resort to self-prescribing practices to reduce costs, including the use of expired antimicrobials or swapping of broad-spectrum antimicrobial drugs, which pose a significant threat to public health [16,17,23,25]. This is especially evident when antimicrobials are accessible in drug stores and black markets without the need for prescriptions [16,17,23,25].

The pervasive use of antifungal agents in healthcare and agriculture has raised concerns regarding the emergence of antifungal resistance in Lebanon. To address this issue, we conducted a survey to identify the antifungal drugs available in both pharmaceutical and agricultural markets. Additionally, we evaluated the current trends of antifungal susceptibility patterns of *Candida* spp. and *Aspergillus* spp. prevalent in Lebanon in order to facilitate effective treatment of fungal infections and reduce the morbidity and mortality associated with these diseases. The results of this study provide valuable bases for developing antifungal stewardship programs and mitigating the emergence of antifungal resistance in the region and beyond.

## 2. Results

### 2.1. Antifungal Use in Human Medicine

We identified a total of 18 antifungal drugs belonging to four different classes. The drugs were classified as follows: (1) azoles (clotrimazole, miconazole, sertaconazole, fluconazole, econazole, omoconazole, fenticonazole, ketoconazole, posaconazole, itraconazole, isoconazole, and voriconazole), (2) allylamines (terbinafine), and (3) echinocandins (caspofungin, anidulafungin, and micafungin), and (4) polyenes (amphotericin-B liposome and nystatin) (Table 1). These drugs are primarily used to treat various fungal infections, including candidiasis (cutaneous, vulvovaginal, or oropharyngeal), dermatophyte infections, and other fungal diseases like non-invasive aspergillosis (treated with azoles), invasive aspergillosis (treated with echinocandins), onychomycosis (treated with allylamines), and pityriasis (treated with sertaconazole). The recommended dosage for these drugs typically ranges from once to twice daily, depending on many factors such as age, weight, infection site, and the size of infected area.

In human medicine, antifungal drugs are either locally manufactured in Lebanon or imported from various European countries and Japan, and they can be administered topically, orally, or intravenously. Most of the drugs are available in both topical and oral forms and can be easily obtained from pharmacies without prescriptions. However, drugs administered intravenously, such as caspofungin, anidulafungin, micafungin, and amphotericin-B liposome, are strictly reserved for hospital use and are not available in pharmacies.

**Figure 1:**
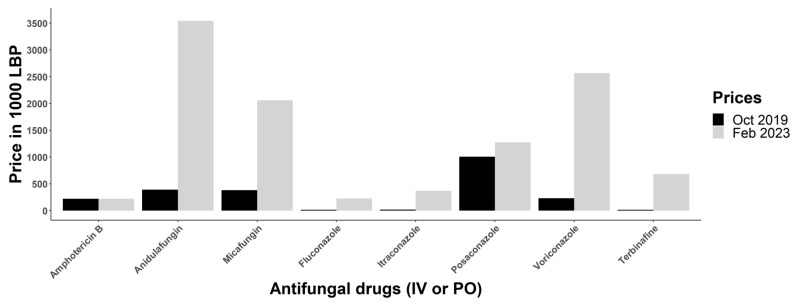
Increase in the prices of available antifungal drugs between October 2019 and February 2023. Before the economic crisis, 1000 Lebanese pounds (LBP) was equivalent to 0.67 USD. Currently, 1000 LBP is equivalent to ∼0.01 USD. All included antifungals are PO (by mouth) or IV (intravenous).

In response to the economic crisis in Lebanon, the Ministry of Public Health initially subsidized a range of essential medications, including antifungal drugs, to ensure patients had access to affordable drugs. However, as the crisis persisted, the government was forced to remove or drastically reduce these subsidies, resulting in a significant increase in drug prices across the country. To assess the impact of these changes on the availability and affordability of antifungal drugs in Lebanon, we evaluated the market prices of antifungal medications that are important in human medicine. We observed that the prices of the most used antifungal drugs significantly increased, by 3 to 12 times in comparison to the pre-crisis cost in some instances, from October 2019 to February 2023 (Figure 1).

### 2.2. Antifungal Use in Agriculture and Veterinary Medicine

We found that six fungicides are currently available in agricultural drug stores and used in Lebanon. Specifically, the highest number of antifungals present in the Lebanese agricultural drug stores belonged to triazoles (difenoconazole, penconazole), followed by pyrimidine derivatives (azoxystrobin, pyrimethanil), thioureas (thiophanate methyl), and copper (copper oxychloride). In addition, three drugs that are widely used in animals without the requirement of a veterinarians’ prescription were found. These drugs belong to different classes of antifungals, including pyrimidine derivatives (diazinon), polyenes (nystatin), inorganic sulfurs (calcium polysulfide), and carboxylic acid amides. (Table 2). The drugs are imported from the United States, Europe, Egypt, Malaysia, India, and China.

Most of the antifungals used to prevent fungal infections in plants and animals are systemic fungicides. Our data revealed six systemic fungicides. For example, difenoconazole, penconazole, pyrimethanil, nystatin, and calcium polysulfide are among the systemic fungicides detected (Table 2). Preventive fungicides are also used in Lebanon, where we found one drug available in agricultural pharmacies (copper oxychloride). Our findings also indicate that there is one preventive and curative fungicide in veterinary clinics and agriculture drug stores (thiophanate methyl). Moreover, a systemic, preventive, and curative fungicide (azoxystrobin + difenoconazole) is available in agriculture drug stores (Table 2).

### 2.3. Antifungal Resistance in Human Medicine and Environment

We screened all available studies (n = 15): 13 (87%) focused on *Candida*, and the remaining two (13%) were on *Aspergillus*. These investigations primarily adopted a cross-sectional approach, complemented by two case reports, one prospective and two retrospective studies. Overall, our analysis revealed a limited number of well-designed national studies (i.e., only one of the 15 studies was nationwide), the non-use of reference susceptibility testing methods in most clinical laboratories, and the absence of epidemiological tracking and national reports.

Assessing the available data, our findings highlighted that *Candida* and *Aspergillus* isolates exhibited a notable resistance to almost all antifungal agents (Table 3). We found that *Candida albicans* isolates were resistant to fluconazole (2% to 33% of isolates tested), ketoconazole (6% to 19%), itraconazole (12.1% to 44%), posaconazole (20.7%), voriconazole (2.5% to 13.8%), micafungin (13.8%), anidulafungin (41.4%), amphotericin-B (0% to 13.8%), and flucytosine (6%). We also found moderately significant resistance rates to antifungal drugs in *Candida glabrata* and *Candida tropicalis*. Specifically, fluconazole resistance ranged from 22.2% to 100% in *C. glabrata* and 16.4% to 95.7% in *C. tropicalis*, while ketoconazole resistance affected 0% of *C. glabrata* isolates and 67% of *C. tropicalis* isolates. Itraconazole resistance was identified in 16.7% to 70.5% of *C. glabrata* isolates and 40% to 67% of *C. tropicalis* isolates, while 11% of *C. glabrata* and 50% to 96.1% of *C. tropicalis* were posaconazole-resistant. Voriconazole resistance was found in 25% to 33% of *C. glabrata* and 0% to 10.5% of *C. tropicalis* isolates. Additionally, *C. glabrata* showed resistance to micafungin (1.5% to 16.7%) and anidulafungin (0% to 61.1%), while 0% to 10% of *C. tropicalis* were resistant to amphotericin-B. Flucytosine resistance was not noted in *C. glabrata* or *C. tropicalis* isolates. Moreover, an important antifungal drug resistance was identified in *Candida parapsilosis* and *P. kudriavzevii*, where 0% to 93.2% and 68% to 100% were resistant to fluconazole, 0% to 75% and 0% to 20% were resistant to itraconazole, 8.3% to 100% and 0% were resistant to posaconazole, 0% to 41.7% and 20% were resistant to micafungin, 0% to 33.3% and 20% were resistant to anidulafungin, and 0% to 8.3% and 0% to 60% were resistant to amphotericin-B, respectively. It is worth mentioning that all tested isolates were susceptible to voriconazole.

Regarding *Kluyveromyces marxianus* (formerly known as *Candida kefyr*), 41.6% of the isolates were resistant to fluconazole, 33.3% were resistant to itraconazole, 50% were resistant to miconazole, and 33.3% were resistant to micafungin; all isolates were susceptible to voriconazole, posaconazole, anidulafungin, and amphotericin-B. The emergent *Candida auris* was frequently reported in different hospitals during the COVID-19 pandemic. *C. auris* circulating in Lebanon consistently demonstrated resistance to fluconazole (100%) and amphotericin-B (100%) and susceptibility to echinocandins (0%). Resistance to voriconazole was observed in 3% of the *C. auris* isolates.

Our analysis showed also a concerning increase in multidrug-resistant *Aspergillus* isolates in Lebanon. Specifically, 32.5% of *Aspergillus niger* isolates were resistant to itraconazole, followed by 25% to posaconazole, 12.5% to voriconazole, and 20% to amphotericin-B. Furthermore, 20% of *Aspergillus flavus* isolates were resistant to itraconazole, along with 30% to posaconazole, 40% to voriconazole, and 70% to amphotericin-B. However, lower resistance rates were observed in *Aspergillus fumigatus* isolated from both clinical [24] and environmental [26] settings.

**Table 3:** Distribution and prevalence of antifungal resistance among common *Candida* and *Aspergillus* species in clinical and non-clinical settings in Lebanon.

Study Period	Study Design	Sample Type (n)	Species (n)	Susceptibility Testing Method	Prevalence of Antifungal Resistance (%) ^¶^											Ref.
					FLZ	KTZ	ITZ	PCZ	VRZ	MIZ	CPF	MCF	ADF	AMB	FLU	
2020–1	Retrospective case control	Urine (n = 20); deep tracheal aspirates (DTA) (n = 26); blood (n = 8); wounds (n = 4)	*Candida auris* (n = 58)	E-test; Vitek-2	100				3		0	0		100		[27]
2021	Case report	Blood (n = 2); urine (n = 2); DTA (n = 2)	*Candida duobushaemulonii* (n = 1)	Vitek-2					R					R	S	[28]
2021	Cross-sectional	Peripheral blood (n = 4); central line catheter (n = 1); DTA (n = 12); Urine (n = 8); skin (n = 2); bronchoalveolar lavage (BAL) (n = 1)	*C. auris* (n = 28)	Vitek-2	100		25		3		0	0		100		[29]
2020	Case report	Blood (n = 2); urine (n = 3); DTA (n = 10); skin (n = 1)	*C. auris* (n = 14)	E-test	100						0	0		100		[30]
2014–9	Cross-sectional	Urine (n = 22); skin (n = 26); vaginal swab (n = 18); ear, nose, and throat (ENT) (n = 3); stool (n = 5); lower respiratory tract (n = 11); pus (n = 3); catheter (n = 2); blood (n = 1); gastric fluid (n = 1); cerebrospinal fluid (n = 1)	*Candida albicans* (n = 29)	Micro-broth dilution	24.1		24.1	20.7	13.8			13.8	41.4	13.8		[31]
			*Candida glabrata* (n = 18)		22.2		16.7	11	33			16.7	61.1	11.1		
			*Candida parapsilosis* (n = 12)		8.3		75	8.3	0			41.7	33.3	8.3		
			*Candida tropicalis* (n = 10)		20		40	50	0			20	60	10		
			*Kluyveromyces marxianus* (n = 6)		0		0	0	0			33.3	0	0		
			*Pichia kudriavzevii* (n = 5)		100		20	0	0			20	20	60		
2016–8	Cross-sectional	Urine (n = 393), vaginal swabs (n = 147), [sputum, blood, cerebrospinal fluid (CSF), miscellaneous] (n = 460)	*C. glabrata* (n = 408)	Micro-broth dilution	100			NA	NA			1.5	0	0		[32]
			*C. tropicalis* (n = 231)		95.7			96.1	0			NA	0	0.4		
			*C. parapsilosis* (n = 103)		93.2			100	0			0	0	0		
			*P. kudriavzevii* (n = 35)		NA			NA	NA			NA	NA	NA		
			*K. marxianus* (n = 72)		NA			NA	NA			NA	NA	NA		
2019	Cohort prospective	Water samples collected across Lebanon (n = 84)	*K. marxianus* (n = 12)	Disc diffusion	41.6		33.3			50						[33]
2015–6	Cross-sectional	Vaginal discharge (n = 258)	*C. albicans* (n = 40)	E-test	10		12.5		2.5					2.5		[34]
2014	Cross-sectional	Stool (n = 41)	*C. glabrata* (n = 6)	Fungitest^™^ (BioRad^®^)	0	0	0			0				0	0	[35]
			*C. albicans* (n = 2)		0	0	0			0				0	0	
			*C. parapsilosis* (n = 1)		S	S	S			S				S	S	
			*C. tropicalis* (n = 1)		S	S	S			S				S	S	
2005–14	Cohort retrospective	Urine (28%); blood (25%); respiratory tract (19%); fluids (15%); wounds/abscesses (11%); catheters (2%)	*C. albicans* (n = 68)	E-test	2		44		4.5					0		[36]
			*C. tropicalis* (n = 72)		16.4		55.5		10.5					0		
			*C. glabrata* (n = 46)		28.7		70.5		25					0		
			*C. parapsilosis* (n = 10)		4.7		25		0					0		
			*P. kudriavzevii* (n = 8)		68		0		0					0		
2010–1	Cross-sectional	Urine (n = 31); sputum (n = 23); tracheal aspirates (n = 12); BAL (n = 11); body fluids (n = 3); abscesses (n = 2); pus swabs (n = 2); abdominal swab (n = 1)	*Candida* spp. (n = 83) *C. albicans* (n = 69)	E-test	69.5			63.5					11.7	37.6		[37]
			*C. glabrata* (n = 8), and													
			*C. tropicalis* (n = 6)]													
2009	Cross-sectional	Sputum (n = 35); stool (n = 24); urine (n = 17); DTA (n = 9); vagina (n = 8); bronchial alveolar wash (n = 8); wound (n = 5); abdominal fluid (n = 2); tooth abscess (n = 1); pus (n = 1); penile swab (n = 1); peritoneal fluid (n = 1); nail (n = 1); blood (n = 1); Jackson–Pratt drainage (n = 1); cerebrospinal fluid (n = 1)	*C. albicans* (n = 116)	E-test; micro-broth dilution	5.2	6	12.1		7.8		0			1.7		[38]
1995–6	Cross-sectional	Respiratory (n = 35); urine (n = 16); blood (n = 4); wound (n = 8); ear (n = 3), catheter (n = 2); abdominal fluid (n = 2)	*C. albicans* (n = 48)	E-test	33	19	21							0	6	[39]
			*C. tropicalis* (n = 12)		33	67	67							0	0	
			*C. parapsilosis* (n = 6)		0	0	0							0	0	
			*C. glabrata* (n = 2)		0	0	0							0	0	
			*P. kudriavzevii* (n = 2)		100	0	0							0	100	
2022	Cross-sectional	Plant beds (n = 67); hospital flowers (n = 5); construction sites (n = 25); flowers beds (n = 50); parks (n = 32); green houses (n = 28); hospital plant beds (n = 6); agriculture fields (n = 49)	*Aspergillus fumigatus* (n = 26)	Micro-broth dilution			0	0	0							[26]
			*Aspergillus neoellipticus* (n = 1)				S	S	S							
			*Aspergillus fischeri* (n = 1)				S	R	S							
2011–9	Cross-sectional	Ears (n = 60); respiratory (n = 8); nails (n = 5)	*Aspergillus niger* (n = 40)	Micro-broth dilution			32.5	25	12.5					20		[24]
			*Aspergillus flavus* (n = 20)				20	30	40					70		
			*Aspergillus tubingensis* (n = 4)				50	50	25					25		
			*A. fumigatus* (n = 3)				33.3	33.3	0					0		
			*Aspergillus terreus* (n = 3)				33.3	33.3	33.3					100		
			Other *Aspergillus* (n = 3)				66.7	66.7	66.7					66.7		

FLZ, fluconazole; KTZ, ketoconazole; ITZ, itraconazole; PCZ, posaconazole; VRZ, voriconazole; MIZ, miconazole; CPF, caspofungin; MCF, micafungin; ADF, anidulafungin; AMB, amphotericin-B; FLU, flucytosine; NA, Information not available. ^¶^ The prevalence of antifungal resistance is shown as R/S when there was only one isolate. We only included the most identified *Candida* and *Aspergillus* species, including *Candida albicans*, *Candida auris*, *Candida duobushaemulonii*, *Candida glabrata*, *Candida parapsilosis*, *Candida tropicalis*, *Pichia kudriavzevii*, *Kluyveromyces marxianus*, *Aspergillus fumigatus*, *Aspergillus neoellipticus*, *Aspergillus fischeri*, *Aspergillus niger*, *Aspergillus flavus*, *Aspergillus tubingensis*, and *Aspergillus terreus*.

## 3. Discussion

The inappropriate and unsupervised use of antifungals in Lebanon appears to be a growing public health concern with significant implications associated with the emergence of multidrug-resistant fungal pathogens, patient outcomes, and healthcare and agricultural systems. Our findings showed an increasing trend in antifungal resistance, emphasizing an urgent need to address this critical issue promptly and effectively at the human–animal–environment continuum. Addressing this burden requires a comprehensive approach that combines various aspects of the One Health ethos, from surveillance to outreach, targeting the proper use of antifungals, appropriate diagnostics, infection control, and patient management.

Antifungal use in the community in Lebanon increased by 88% from 2004 to 2018, as measured by the number of defined daily doses, with the highest consumption level noted in 2017 [40]. Triazoles (73.6%) were the most consumed antifungals, including fluconazole (21%), itraconazole (12.5%), voriconazole (0.15%), and posaconazole (0.02%). Echinocandins and amphotericin-B were the least used, with a combined consumption of less than 0.03% [40]. Alarmingly, no data are available on the use of antifungals in animals and in the agriculture sector. Despite this, the increasing trends in resistance have been commonly associated with the use of antifungal agents without a prescription in humans, animals, and agriculture [24,31]. To date, the over-the-counter use of antifungal drugs remains a regular habit among resident communities in Lebanon, including farmers and the general population [25].

Intravenous antifungals are often appropriately used because they are typically reserved for in-hospital use. However, the COVID-19 pandemic has brought about a concerning surge in fungal infections within intensive care units of healthcare facilities, resulting in an increased reliance on broad-spectrum antifungals [41]. This was precipitated further by the ongoing Lebanese economic crisis [17]. Indeed, due to the shortage of suitable diagnostics for antimicrobial resistance, healthcare providers have been forced to make critical treatment decisions without access to essential information, further complicating patient care and potentially promoting the selection of resistant fungi [23,42]. Prolonged or inappropriate use of these medications selectively favors the emergence of non-*albicans* species that inherently possess higher resistance to these treatments [43]. Notably, since the onset of the COVID-19 pandemic, the incidence of *C. auris* infections in critically ill patients in healthcare settings has been steadily increasing across hospitals in Lebanon [27,29,30]. The first reported outbreak of *C. auris* occurred during the pandemic (October–December 2020) at the American University of Beirut Medical Center [30], posing serious public health concerns due to its multidrug-resistant properties and elevated transmission ability. Albeit underestimated due to the lack of reliable identification tools in most Lebanese clinical laboratories and the absence of a national reference center for mycoses and antifungals, the characterization of the current circulating isolates indicated a uniform resistance to fluconazole and amphotericin-B [29]. Furthermore, as witnessed worldwide, we observed an evident shift of *C. albicans* toward less susceptible non-*albicans Candida* (i.e., mainly *C. glabrata* and *C. tropicalis*) over the last two decades [31,44], which also raises concerns about the potential impact on patient outcomes and treatment effectiveness. This highlights the importance of reliable diagnostics and prudent antifungal drug administration to mitigate the development and spread of resistant fungal pathogens.

Little information is available in the literature about antifungal susceptibility in the Middle Eastern and North African (MENA) countries, but a few previous studies reported varied rates of resistance to antifungals. Overall, the data from Lebanon showed that *Candida* and *Aspergillus* isolates exhibited a notable level of resistance to almost all antifungal drugs compared to neighboring countries. In Lebanon, fluconazole resistance ranged from 2% to 33% in *C. albicans*, 22.2% to 100% in *C. glabrata*, and 16.4% to 95.7% in *C. tropicalis*. In Tunisia, resistance to fluconazole was observed in 63 % and 16% of *C. albicans* and *C. glabrata* isolates from vaginal swab samples of pregnant women, respectively [45]. Moreover, numerous sporadic cases and outbreaks caused by *C. auris* have been recently reported in the MENA region, including Lebanon [27,29,30,46,47,48]. Other reports on *Aspergillus* showed differing frequency of azole resistance as follows: 31.5% were resistant in Lebanon [24], 30.6% were resistant in Iran [49], 3.3–25% were resistant in Turkey [50,51,52], 4.1% were resistant in Tunisia [53], 2.9% were resistant in Qatar [54], and 0% were resistant in Kuwait [55].

The majority of antifungals used in agriculture are classified as preventive fungicides (i.e., prevent crop loss and increase agricultural yield) [56]. This has significant implications, because these treatments tend to accumulate within plant tissues and can affect the environment, potentially promoting the development of resistant fungal pathogens of clinical and veterinary interests. The transmission of resistant fungi can occur through spore inhalation, direct contact with treated plants, or the handling of other contaminated agricultural items such as soils and tools. Importantly, it should not be neglected that the rise in antifungal resistance threatens global food security, because uncontrollable fungal infections can devastate crops, increasing poverty, malnutrition, and disease, especially in disenfranchised communities. Taken together, our observations highlight the importance of exploring robust stewardship programs and alternative approaches to manage fungi in medical and agricultural practices. This could precipitate in minimizing the risk of resistance development and subsequent implications for public health and food security.

The study presented here had a few limitations. Our approach did not encompass the consideration of non-official antifungals that might have been illicitly introduced into Lebanon or brought in by the diaspora to support local communities. Additionally, the available data obtained previously in Lebanon addressed only the epidemiology of clinical strains of *Candida* and *Aspergillus*; thus, the epidemiological significance of the existing findings remains to be confirmed. Furthermore, the lack of reliable identification tools in most of the available studies hindered attempts to explore genetic profiles of isolates and identify underlying resistance mechanisms. To better understand the dissemination of these fungi and the risk factors associated with the spread of antifungal resistance, it is necessary to conduct further prospective multicentric studies involving a substantial number of clinical and non-clinical isolates.

## 4. Materials and Methods

This study was conducted between January and February 2023 to identify antifungal drugs that are used in human and animal medicine in Lebanon. The Lebanese Ministry of Public Health, as well as major pharmacies, veterinary clinics, and agriculture markets located in Tripoli and Beirut, provided the primary data sources. The study collected comprehensive data on the available antifungals, including their antifungal class, brand name, active molecules, administration routes, availability in pharmacies, veterinary and agricultural stores, and healthcare settings. The study also recorded the usual recommended treatment regimens, treatment indications, and manufacturing country of origin. In addition, the current price of the antifungal drugs of clinical interest was compared to the price before the economic collapse (October 2019) in Lebanon.

Moreover, we searched PubMed, Science Direct, and Google Scholar databases for studies on fungal and antifungal resistance between 1998 and 2023. We selected a word combination that included “yeast”, “fungal”, “fungal infection”, “fungi”, “antifungal”, “antimicrobial resistance”, “*Candida*”, “aspergillosis”, “azole”, and “One Health”. We reviewed all relevant publications. Original publications in English or French that were indexable and of any epidemiologic methodology, sampling technique, or type (case report, longitudinal, case–control, or cross-sectional) were included. All research that published original data on antifungal resistance in Lebanon was considered for inclusion in the study.

## 5. Conclusions

As *Candida* and *Aspergillus* species continue to challenge healthcare settings, implementing robust One Health programs that rely on reliable diagnostic tools is essential to monitor the prevalence of these species and assess their susceptibility patterns. Indeed, using the micro-broth dilution technique following the international guidelines has become crucial in patient management and resistance surveillance. Additionally, enhancing awareness and strengthening infection control measures, such as strict adherence to hand hygiene protocols and proper disinfection practices, are required to limit the spread of these resilient pathogens. Lastly, we recommend the establishment of national reference centers for invasive mycoses and antifungal resistance and new regulations to prevent the inappropriate use of antifungal drugs in humans and agriculture.

## Figures and Tables

**Table 1:** Summary of available antifungal drugs used in human medicine in Lebanon.

Antifungal Class	Brand Name	Active Drug	Route	Available in Pharmacies	Manufacturer Origin	Recommended Dose	Indication
Azoles	Canesten	Clotrimazole	Topical	Yes	Germany	5 g daily	Cutaneous candidiasis, dermatophyte infections
	Daktarin	Miconazole	Topical	Yes	Belgium	5 g daily	Cutaneous or oropharyngeal candidiasis
	Dermofix	Setraconazole	Topical	Yes	Spain	Variable ^^^, daily or BID	Cutaneous candidiasis, dermatophyte infections, pytiriasis
	Diflucan	Fluconazole	PO *, IV	Yes	France	200 mg daily ^+^	Candidiasis, cryptococcal meningitis, coccidiomycosis
	Econaz	Econazole	Topical	Yes	Lebanon	Variable ^^^, daily or BID	Cutaneous candidiasis, dermatophyte infections
	Fongamil	Omoconazole	Topical	Yes	France	Variable ^^^, daily or BID	Cutaneous candidiasis, dermatophyte infections
	Lomexin	Fenticonazole	Topical	Yes	Lebanon	5 g BID	Vulvovaginal candidiasis
	Nizoral	Ketoconazole	Topical	Yes	Belgium	Variable ^^^, twice weekly	Tinea capitis
	Noxafil	Posaconazole	PO	Yes	Netherlands	300 mg daily, 300 mg BID	Aspergillosis, candidiasis
	Sporanox	Itraconazole	PO	Yes	Lebanon	200 mg BID	Aspergillosis, candidiasis
	Travogen	Isoconazole	Topical	Yes	Italy	Variable ^^^, daily	Cutaneous candidiasis, dermatophyte infections
	V-fend	Voriconazole	PO, IV	Yes	PO: Germany, IV: France	200 mg BID	Candidiasis, candidemia, aspergillosis, *Scedosporium* infection, *Fusarium* infection
Allylamines	Lamisil	Terbinafine	PO, Topical	Yes	PO:Germany, Topical: Switzerland	PO: 250 mg daily. Topical: variable ^^^. daily or BID	Onychomycosis
Echinocandins	Cancidas	Caspofungin	IV	No ^§^	France	70 mg once then 50 mg daily	Invasive candidiasis, invasive aspergillosis, neutropenic fever
	Ecalta	Anidulafungin	IV	No ^§^	Belgium	200 mg once then 100 mg daily	Invasive candidiasis, invasive aspergillosis, neutropenic fever
	Mycamine	Micafungin	IV	No ^§^	Japan	100 mg daily	Invasive candidiasis, invasive aspergillosis, neutropenic fever
Polyenes	Ambisome	Amphotericin-B liposome	IV	No ^§^	Ireland	3–10 mg/kg	Invasive candidiasis, invasive aspergillosis, neutropenic fever, cryptococcal meningitis,
	Medistan	Nystatin	Topical	Yes	Lebanon	40,000 to 60,000 U QID	oropharyngeal candidiasis

PO *, per os (by mouth); IV, intravenous; BID, twice daily; QID, four times daily. ^^^ Dose varies according to the size of infected area. ^+^ This dosage regimen is used for oropharyngeal candidiasis. ^+^A higher dosing regimen might be needed in certain infections, e.g., cryptococcal meningitis. ^§^ Only available in hospitals.

**Table 2:** Available antifungal drugs used in agriculture and veterinary applications in Lebanon.

Antifungal Class	Use	Brand Name	Active Molecule (Formulation)	Treated Animals/Plants	Treatment Indications	Withdrawal Time	Manufacturer Origin
Triazoles	Systemic fungicide	Sakay (liquid formulation)	Difenoconazole (250 g in 1 L)	Winter wheat, oilseed rape, brussels sprouts, cabbage, broccoli/calabrese, cauliflower	Broad spectrum	7 days	Malaysia
Copper	Preventive fungicide	Fungatox (powder)	Copper oxychloride (2.50 g in 1 L)	Onion, garlic, apple, pear, tonsils, grapes, cowpeas, peas, olives, citrus, chickpeas, spinach	*Alternaria* spp., *Phytophthora* spp.	3–15 days	India
Triazoles	Systemic fungicide	Topas 100 EC (liquid formulation)	Penconazole (100 g in 1 L)	Apple tree, pear tree, quince, strawberry, cucumber, melon, zucchini, eggplant, peppers, tomato	Broad spectrum	3–35 days	Switzerland
Aryloxypyrimidine	Preventive, systemic and curative fungicide	Amistar top (liquid formulation)	Azoxystrobin (200 g in 1 L) + difenoconazole (125 g in 1 L)	Barley, grapes, maize, onions, peas, potatoes, ryegrass seed crops, sweetcorn, field tomatoes, wheat	Broad spectrum	3–7 days	United Kingdom
Thioureas	Preventive and curative fungicide	Gypson (powder)	Thiophanate methyl (70% WP)	Apples, pears, small grains, cucurbits, peanuts, garlic, onions, almonds, potatoes, tobacco, vegetables, bananas, figs, brassicas, citrus, and strawberries.	Broad spectrum	3–7 days	China
Anilinopyrimidine	Systemic fungicide	Kuile (liquid formulation)	Pyrimethanil (40% SC)	Grapes, tomatoes, strawberries	*Botrytis cinerea*	3–7 days	China
Polyene	Systemic fungicide	Nystatin (tablet or oral liquid)	Nystatin	Cats, dogs, reptiles, birds	*Candida*, fungal infections in the mouth or gastrointestinal tract	1 to 2 days	
Inorganic sulfur	Systemic fungicide	Lime sulfur (cream)	Calcium polysulfide (calcium hydroxide and sulfur) (0.16 g in 100 g)	All animals	Dermatophyte infections	1 time daily for 1 week, then 2 times per week for 2 more weeks	United States
Carboxylic acid amides	Systemic fungicide	Anti-mycotoxins (powder)	1,3-Beta glucan (125 mg in 1 kg) + mannan oligosaccharide (90 mg in 1 kg) + formic acid (100 mg in 1 kg) + acetic acid (100 mg in 1 kg) + propionic acid (100 mg in 1 kg)	Birds	*Aspergillus flavus*, *Aspergillus parasiticus*, *Penicillium* spp., *Aspergillus* spp., *Fusarium* spp., *Byssochlamys* spp.	None	Egypt

EC, emulsifiable concentrate; WP, wettable powder; SC, suspension concentrate.

## Data Availability

None to declare.
